# Comment on “Computer algorithm can match physicians’ decisions about blood transfusions”

**DOI:** 10.1186/s12967-021-02841-2

**Published:** 2021-04-28

**Authors:** Sander De Bruyne

**Affiliations:** Department of Diagnostic Sciences, Ghent University, 9000 Ghent, Belgium

To the Editor,

I read with interest the article by Yao et al. [1], entitled “Computer algorithm can match physicians’ decisions about blood transfusions”. In this study, the authors designed a multilayer perceptron neural network to predict the appropriateness of intra-operative blood transfusion cases. Of the 4946 patients who received intra-operative blood transfusions, 3604 cases were classified as appropriate and 1342 as inappropriate by expert anesthesiologists based on guidelines of the World Health Organization. The authors claimed that the neural network trained on these data achieved a promising 96.8% accuracy rate in matching human judgement: 99% of the computer’s decisions matched the experts on the appropriate cases and 90.9% matched on the inappropriate ones. While the concept of the study is interesting and while I believe that machine learning models could be useful tools in the screening for blood transfusion overconsumption on a larger scale, the reported accuracy results should be interpreted with caution. One of the key concepts in the creation of reliable machine learning models is to split the dataset into separate training, validation and test sets. While a training set can be seen as a subset of data employed for fitting the model’s parameters, a validation set can be defined as a set of examples to tune the parameters of the model such as the selection of an optimal number of hidden layers in a neural network. However, it has to be mentioned that *k*-fold cross-validation can also be regarded as a valuable alternative for traditional validation sets. After training and validation, it is common practice to evaluate the model’s performance on unseen (i.e. independent) test data samples in order to obtain a reliable estimation of the generalization error, which is the error rate on the prediction of new data [2]. In this study, no splitting procedure is described in the materials and methods section and it is stated that all 4946 data entries were inputted to the model. Moreover, as can be derived from the supplementary python script, it seems that the model was trained, validated and tested on exactly the same data entries. Therefore, the reported accuracies in this study are likely biased, have to be interpreted as the results of a potentially overfitted model and cannot be perceived as being per definition valid for independent data samples.

## Data Availability

Not applicable.
